# Patient preferences regarding treatment options for Waldenström's macroglobulinemia: A discrete choice experiment

**DOI:** 10.1002/cam4.5080

**Published:** 2022-07-26

**Authors:** Karima Amaador, Pythia T. Nieuwkerk, Monique C. Minnema, Marie José Kersten, Josephine M. I. Vos

**Affiliations:** ^1^ Amsterdam UMC, University of Amsterdam, Department of Hematology Cancer Center Amsterdam Amsterdam The Netherlands; ^2^ Lymphoma and Myeloma Center Amsterdam (LYMMCARE) Amsterdam The Netherlands; ^3^ Department of Medical Psychology Academic Medical Center Amsterdam The Netherlands; ^4^ Department of Hematology, University Medical Center Utrecht University Utrecht Utrecht The Netherlands

**Keywords:** discrete choice experiment, macroglobulinemia, patient preferences, waldenstrom's

## Abstract

**Introduction:**

Treatment options for Waldenström's Macroglobulinemia (WM) have expanded rapidly in the last decades. However, there is no consensus on a preferred treatment. Therefore, patient preferences become increasingly important in making individualized treatment plans. Still, WM patients' priorities and perspectives regarding their treatment options are unknown. We evaluated treatment preferences of WM patients using a discrete choice experiment (DCE).

**Methods:**

A mixed‐method approach was utilized for identification and selection of attributes/levels. The DCE questionnaire included five attributes: type of agent (targeted versus chemotherapy); frequency and route of administration; 5‐year progression‐free survival (PFS); adverse events; and risk of secondary malignancies. An orthogonal design and a mixed logit panel data model were used to construct choice tasks and assess patient preferences, respectively.

**Results:**

Three hundred thirty WM patients participated in the project. In total, 214 (65%) complete questionnaires were included for data analysis. The 5‐year PFS, followed by risk of secondary malignancies were the most important attributes for making treatment choices. Regarding side effects, patients chose to avoid neuropathy the most compared to nausea/vomiting and extreme fatigue. Patients preferred a fixed‐duration treatment with IV/SC administration at the hospital over a continuous daily oral regimen at home.

**Conclusion:**

These are the first systematic data obtained on WM patient preferences for treatment. The results may help discussions with individual patients about their treatment choices. Also, these data can help design clinical trials in WM and inform health‐care decision‐making regarding outcomes that are most relevant to patients.

## Graphical abstract

When asked about treatment preferences, WM patients prioritize avoiding the risk of secondary malignancies, avoiding peripheral neuropathy as a side effect, prefer targeted therapy over chemotherapy, but prefer fixed‐duration iv/sc over long‐term oral treatment. A treatment regimen that matches all these priorities is currently not available for WM.

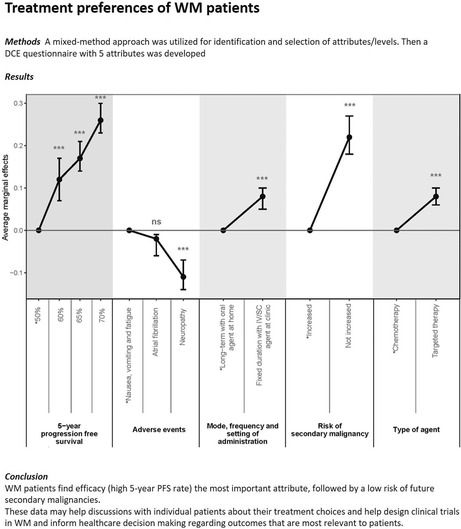

## 1 INTRODUCTION

Waldenström's Macroglobulinemia (WM) is a rare and indolent disease representing approximately 1%–2% of all non‐Hodgkin lymphomas.^1^ WM is incurable with a median age of 65–70 years at diagnosis.^2^ The median overall survival (OS) as reported in 2015 in WM is approximately 7 to 10 years and has possibly further improved since then with the advent of novel therapeutic agents such as BTK inhibitors.^3^, ^4^ The natural disease course of WM is heterogeneous as it varies from an acute presentation requiring immediate therapy to a long asymptomatic course without need for treatment.^5^ However, the majority of WM patients will develop a treatment indication at some time point during the disease course.^6^

The management of WM has evolved in recent years. Rituximab, introduced in the early 2000s, altered the treatment landscape of WM due to its ability to induce a response with low toxicity.^5^ In Europe and more specifically the Netherlands, chemoimmunotherapy is the most commonly applied first‐line systemic treatment in WM, and often comprises a combination of rituximab with an alkylating agent such as cyclophosphamide or bendamustine.^7^, ^8^, ^9^, ^10^, ^11^, ^12^ In more recent years, novel targeted agents like proteasome inhibitors (such as bortezomib) in combination with rituximab and steroids and Bruton tyrosine kinase inhibitors (BTKs) were added to the therapeutic arsenal of WM.^13^, ^14^ Currently available treatments are very different in terms of efficacy, toxicity profile, treatment duration, route of administration, and type of agent (e.g., chemotherapy vs. targeted therapy). Due to the rarity of WM, randomized clinical trials comparing different therapeutic options are scarce. Therefore, there is currently no consensus on a preferred treatment in the first‐line or relapsed setting.

A better understanding of patient treatment preferences can aid physicians and patients in shared decision‐making. Patients who are involved in clinical decision‐making are more likely to express satisfaction with therapy which leads to improved adherence.^15^ In addition, better insight in patients' treatment views could help direct future clinical trials in WM. For instance, preference data can provide valuable information from a patient's perspective to help select clinical trial outcomes that may otherwise be overlooked. Furthermore, these data can also be integrated in health‐care decision‐making, for example, for funding agencies and drug approval.^16^

Discrete choice experiments (DCEs) are being increasingly used to elicit patient preferences in health care.^17^, ^18^ In DCEs, treatments are described by their most important characteristics, also known as attributes (e.g., duration of therapy) with the attributes being specified by several levels (e.g., fixed duration or continuous). DCEs allow for the quantification of the relative importance of these treatment attributes and the trade‐offs that respondents make when faced with a series of choice tasks.^19^

We aimed to assess patient preferences for the currently available WM treatment modalities. In addition, the preferences of WM patients' caregivers/partners were also assessed.

## 2 METHODS

### 2.1 Discrete choice experiment

In the DCE, the respondents were asked to choose between treatment A or treatment B, thereby making trade‐offs between attributes and their levels.

### 2.2 Identifying attributes and levels

**FIGURE 1:**
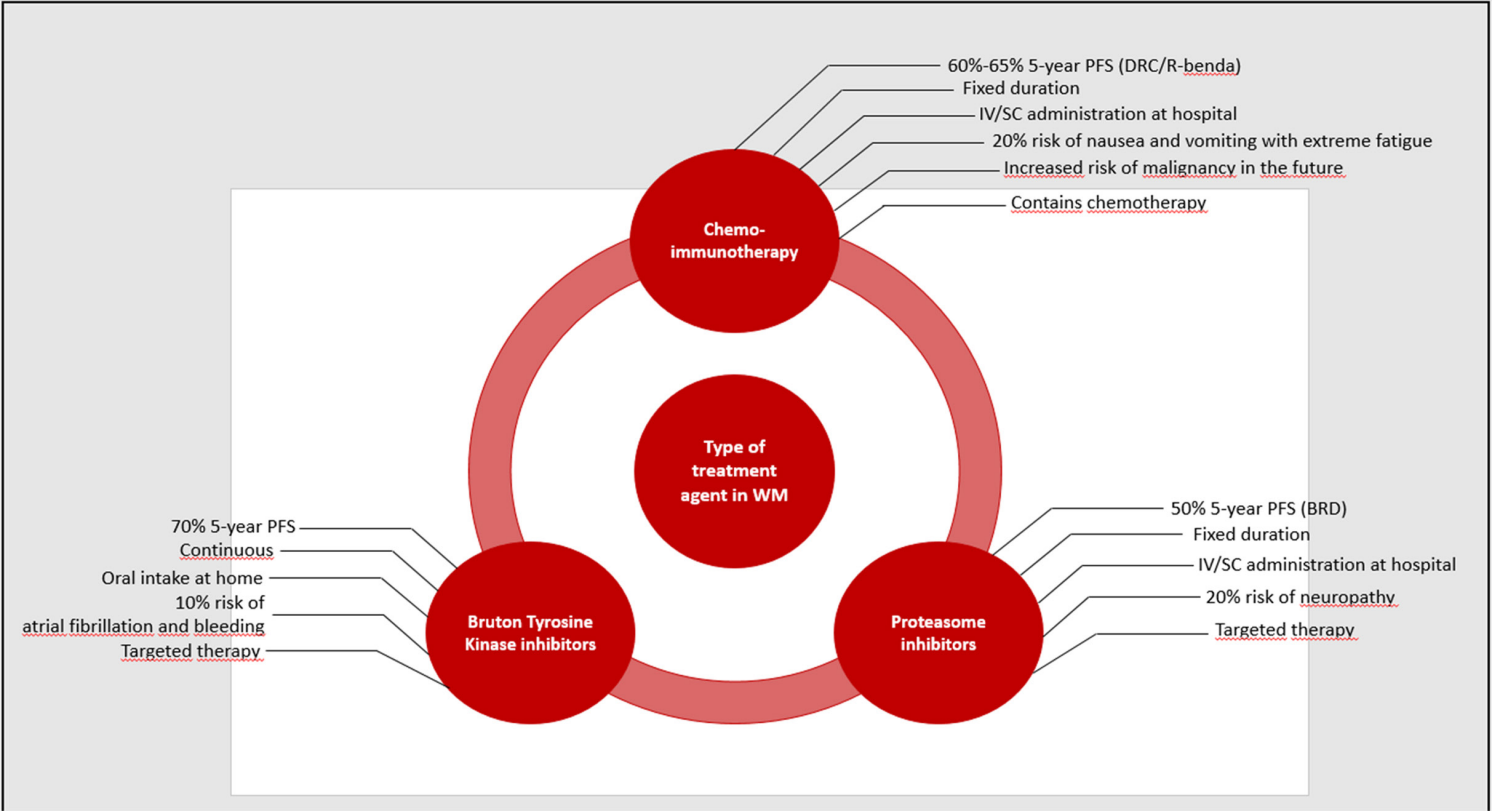
Attributes and levels according to treatment types in Waldenström's Macroglobulinemia.

In this study, a two‐stage design was followed as per the recommendations on how to conduct DCEs in health care.^20^ First, a literature review was conducted to generate an overview of potential attributes and their associated levels describing the currently applied therapeutic regimens in WM. This overview consisted of data from clinical trials and real‐life case series in WM patients and current treatment guidelines. The list of attributes was reviewed by and discussed with WM experts (*JV, MJK*). Subsequently, the list of attributes was presented to a select group of WM patients (*n* = 6) and the attributes were discussed in individual sessions and one group discussion. The discussions included a ranking exercise. The five attributes that were consistently selected as important for patients were progression‐free survival (PFS), dosing, route and setting of administration, adverse events (AEs), risk of secondary malignancy in the future, and type of agent. We, therefore, selected these attributes for the DCE. The selected attributes and associated levels are presented in Table 1. The levels were assigned to the attributes based on the literature review and expert discussions and an overview is presented in Figure 1.

**TABLE 1:** Regimen attributes and levels used in the DCE

Attribute	levels	Characteristic	Described in survey as
Progression‐free survival (PFS)	4	5‐year PFS is: 50% 60% 65% 70%	After 5 years, the disease is still suppressed in.% of the patients
Dosing and administration Frequency of administration Mode of administration Setting for administration	2	Fixed duration vs. continuous Combination of oral, subcutaneous, and intravenous vs. oral only Hospital vs. at home	A fixed‐duration treatment; every 3 weeks for 6 months in the outpatient hospital with subcutaneous/intravenous administration or a combination of the above vs. An at home maintenance treatment in which the medicine has to be taken orally until ineffective or excess symptoms occur.
Adverse events	3	10% risk of atrial fibrillation and increased bleeding risk 20% risk of nausea and vomiting with extreme fatigue 20% risk of peripheral neuropathy	One in 10 patients experience cardiac arrhythmias and/or an increased tendency to bleed vs One in five patients suffers from damage to the nerve endings leading to pain and numbness in the hands and feet. vs. one in five patients experience nausea and vomiting and severe fatigue during treatment
Long‐term side effects: Risk of secondary malignancy in the future	2	Increased Not increased	Risk of other cancers in the future is increased/not increased
Type of agents in regimen	2	Chemotherapy Targeted therapy	Regimen contains: chemotherapy vs. chemotherapy‐free regimen with targeted therapy

### 2.3 Experimental design

**FIGURE 2:**
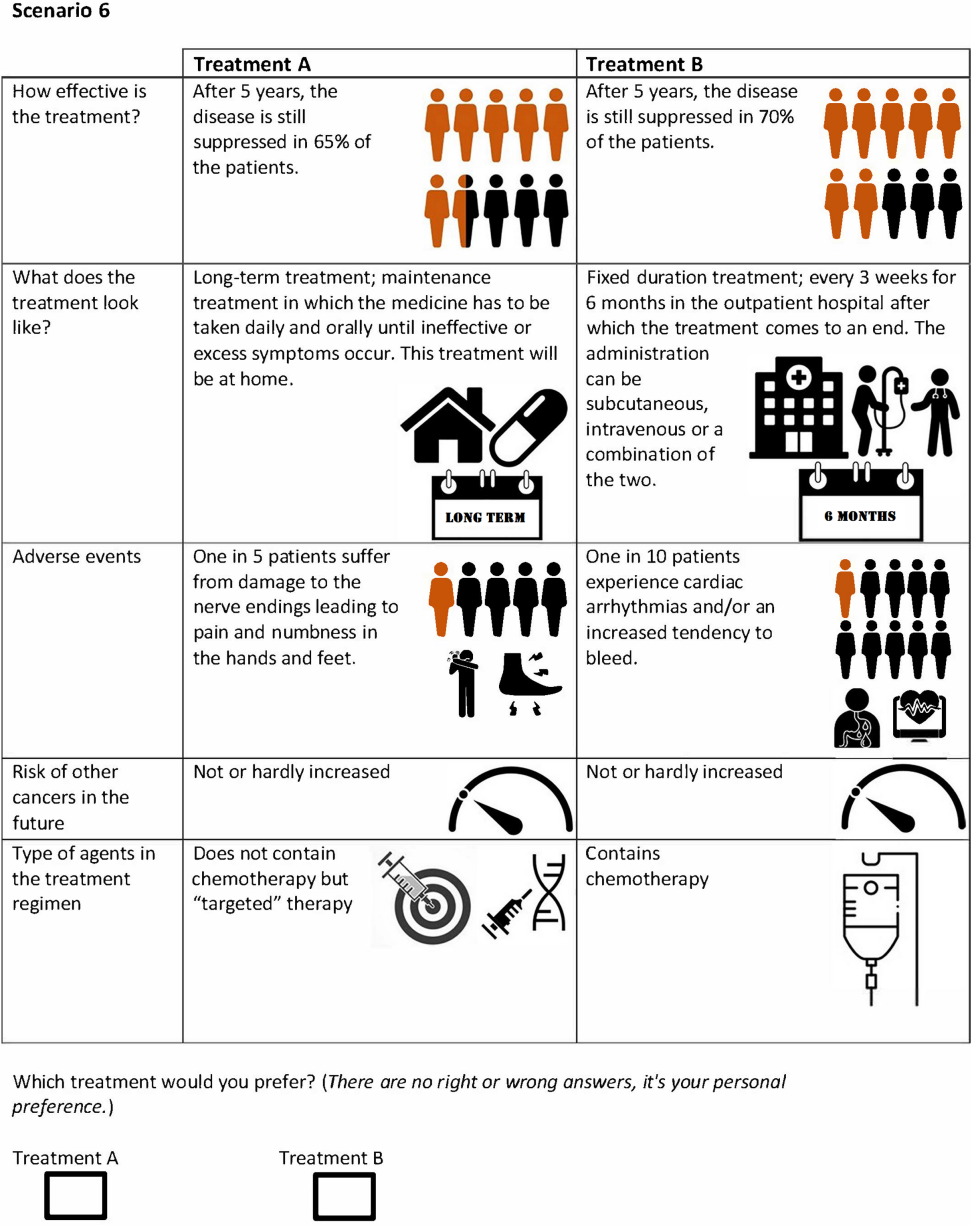
Example of a choice task.

Since it would not be feasible to present the respondents with all possible treatment combinations using the attributes and levels in Table 1, we used an orthogonal design to extract a subset of hypothetical treatments for inclusion in the DCE using R (v3.6.1) software.^20^ By maximizing the D‐efficiency—a summary measure of the variance/covariance matrix—, the precision of the estimated parameters for the subset of choice questions increases. In this study, 16 choice hypothetical but realistic sets were created. The design resulted in a D‐efficiency of 0.88 (the closer this number is to 1, the higher the efficiency). The choice sets were visualized graphically in order to aid the comprehensibility of the attributes and attribute levels. An example of a choice task is shown in Figure 2.

First, a pilot DCE study, which consisted of patient interviews (*n* = 5), was conducted to assess comprehensibility as previously described.^21^, ^22^ The results of this pilot study were used to create the definitive experimental design of the DCE. To avoid unrealistic treatment combinations (e.g., intravenous administration at home), we merged the attributes, mode of administration, frequency of administration, and setting for administration into one attribute.

### 2.4 Questionnaire development

The first part of the survey contained a brief questionnaire on sociodemographic and clinical characteristics such as age, gender, educational level, year of WM diagnosis, treatment history, and current treatment status. The second part contained a thorough explanation of the DCE task, followed by the 16 choice tasks. At the end of the survey, the respondents were asked to rate the difficulty of the choice tasks on a 10‐point scale. The questionnaire was developed in Dutch by an expert in treatment preference assessment (PN) and clinical experts (JV and MJK).

### 2.5 Data collection

Participants were recruited via two routes: an invitation to the anonymous online survey was published on the patient organization (Hematon) website, and in addition, patients diagnosed with WM from the outpatient clinic at Amsterdam University Medical Centers who have indicated their willingness to participate in scientific research, were sent a paper‐based questionnaire including a stamped return envelope. In addition, we also asked patients' caregivers to participate in this survey via the abovementioned website to investigate whether having sufficient knowledge about WM, and lived experience as a caretaker, but not actually having the disease would influence treatment preferences. A separate link containing a slightly modified questionnaire (but with the same set of choice tasks) was provided on the website for patients' caregivers to fill out.

The study protocol was submitted to the Ethical Committee at Amsterdam University Medical Centers, but since the survey was anonymous and did not contain personal data, the need for formal ethical approval and informed consent was waived in accordance with Dutch legislation.

### 2.6 Statistical analysis

The DCE method is based on the random utility theory (RUT).^23^ The DCE was analyzed with a panel mixed logit model accounting for the repeated choice data, that is, 16 choices per respondent, using Stata (version 16.1.). The dependent variable was the binary choice (i.e., treatment A or B), whereas independent variables were the attributes. The panel structure was defined by respondent. All attributes were included as categorical variables. For the efficacy attribute, consisting of the 5‐year progression‐free survival, the reference level was set at the lowest level of 50%. For the attribute side effects, the risk of nausea and vomiting was set as reference category because these are the classical side effects of chemotherapy. All other attributes were dichotomous. The efficacy attribute was included as random effect; the other attributes were included as fixed effects. All models were main effects, that is, no interaction terms were included. We established whether or not the attributes presented in the choice scenarios were significant predictors of patients' treatment preference by examining the statistical significance of the coefficients of the attributes/levels. Coefficients of the attributes and their respective levels were transformed using the “margins” command in Stata to reflect the average marginal effects of the attributes. Average marginal effects indicate the change in the probability of choosing a treatment option, given a change in the level of the respective attribute compared to the reference level. We did this for all attributes to further assess which attributes had the largest impact on treatment preference.

To identify potential patient characteristics that are significantly associated with treatment preference, additional analyses were performed that incorporated the covariates' age, gender, treatment status (wait and see, remission, progression), and educational level into the model.

We repeated the analysis with efficacy included as a continuous variable to be able to calculate marginal willingness to trade efficacy (WTTE) for all attributes/levels. WTTE allows for comparison of the preferences for all attributes, and the WTTE value indicates how much an individual is willing to trade for a one‐unit change in the attribute level. The WTTE is calculated by dividing the coefficients of the various attributes/ levels by the coefficient of efficacy (denominator). Confidence intervals (CI) for WTTE were calculated using a macro in Stata as described by Hole.^24^ Two‐sided *p*‐values <0.05 were considered to indicate statistical significance. All analyses were repeated for the responses from the patients' partners/caregivers.

## 3 RESULTS

### 3.1 Patients' characteristics

The survey was conducted from August to December 2020. A total of 330 online questionnaires and 17 paper‐based questionnaires (from the 38 patients invited per mail) were returned. We excluded 41 and 68 questionnaires because patients completed only demographic questions but not the DCE or because they completed only part of the questionnaire. A total of 214 (65%) complete questionnaires were included for the data analysis. The sociodemographics and clinical characteristics are presented in Table 2. The median age of the respondents who completed the DCE was 67 years (min‐max 29–91 years) and 54% were males. The median time since WM diagnosis was 5.4 years and 56% of respondents were previously treated, 25% were treatment‐naïve, while 19% of the respondents were being treated at the time of the survey. Difficulty of the choice tasks was estimated with an average of 5.4 out of a 10‐point scale by the respondents. The questionnaire for caregivers received 51 responses.

**TABLE 2:** Sociodemographics and clinical characteristics of the respondents

	Patients who completed DCE (*n* = 214)	Patients who completed only demographic questions but not DCE (*n* = 41)	*p* value
Age (years, median [SD; min‐max])	67 (9.8; 29–91)	63 (14.2; 29–91)	0.09
Males, *n* (%)	35/65 (54%)^b^	8/17 (47%)^c^	0.82
Time since diagnosis (years, min‐max)	6.9 (0.1–48)	4.3 (0–20.1)	
High educational level^a^, *n* (%)	115/209 (55%)	16/40 (40%)	0.12
Disease status			0.38
Wait and see	66/211 (31%)	17/37 (46%)	
Remission	83/211 (39%)	11/37 (30%)	
Progression	20/211 (10%)	3/37 (8%)	
Currently treated	42/211 (20%)	6/37 (16%)	
Previously treated at time of completion of the questionnaire			0.31
Yes	122/212 (58%)	17/38 (45%)	
No	60/212 (28%)	15/38 (40%)	

^a^ High educational level defined as completed HBO (Higher Vocational Education) or University.

^b^ Missing: 149.

^c^ Missing: 24.

### 3.2 Patient preferences (DCE)

**FIGURE 3:**
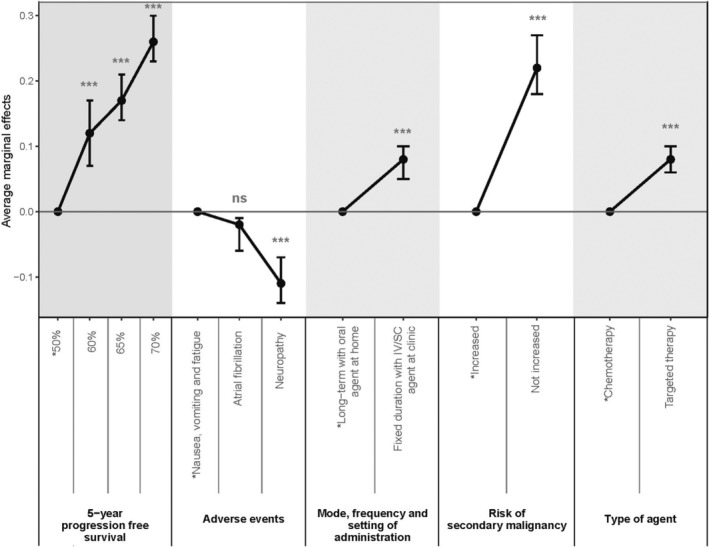
WM patients: Average marginal effects indicating the change in probability of choosing a treatment option if the attribute level was changed from the reference category. The reference categories are depicted with *. The error bars represent the 95% confidence intervals about the point estimate. *p < 0.05, **p < 0.01, ***p < 0.001, ****p < 0.0001.

The average marginal effects of all attributes/levels estimated with the mixed logit model are presented in Figure 3. The average marginal effects of all attributes/levels were statistically significant (*p* < 0.001) indicating that they significantly affected patient preferences, with one exception. The attribute level; ‘atrial fibrillation/increased risk of bleeding’ of the attribute adverse events did not significantly differ from the reference level ‘risk of nausea, vomiting and severe fatigue’ (*p* = 0.1). This indicates that if patients had to choose between the risk of ‘atrial fibrillation/increased risk of bleeding’ compared with the ‘risk of nausea, vomiting and severe fatigue’ they were neutral concerning these two adverse events and this did not significantly influence their choice. The 5‐year PFS, followed by the risk of secondary malignancies in the future were the most important attribute for making treatment decisions. The probability of choosing a treatment option increased with 26% (95% CI: 23% to 30%) if the 5‐year PFS was increased from 50% to 70%. The probability of choosing a treatment option increased with 22% (95% CI: 18% to 27%) if the risk of future secondary malignancies was decreased from a “high risk” to a “low risk”. Regarding the three AEs, patients wanted to avoid neuropathy the most (the probability of choosing a treatment decreased with 11% [95% CI: −14% to −7%] when a treatment increased the risk of neuropathy). For the attribute ‘type of agent’ (targeted therapy vs chemotherapy) the probability of choosing a treatment option when the treatment contained targeted therapy increased with 8% (95% CI: 6% to 10%). Similarly, the attribute ‘dosing and administration’ also resulted in an 8% (95% CI: 5% to 10%) increase of the probability of choosing a treatment option when the treatment comprised a fixed‐duration treatment with an intravenous/subcutaneous (IV/SC) administration at the hospital as opposed to continuous daily oral treatment at home.

### 3.3 Willingness to trade efficacy for other attributes

**FIGURE 4:**
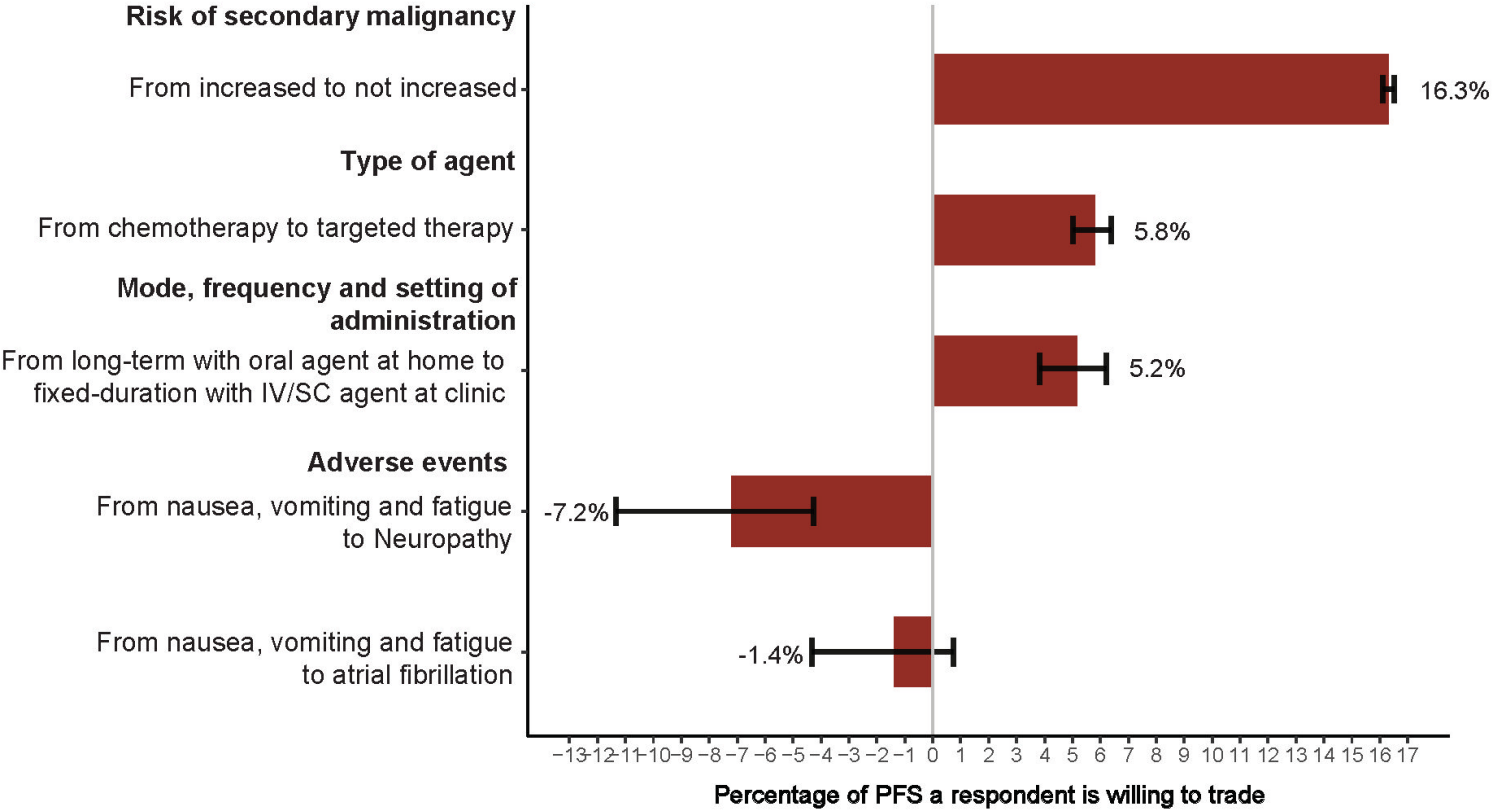
Willingness to trade efficacy for WM treatment attributes. The error bars represent the 95% confidence intervals about the point estimate.

Patients were willing to trade treatment efficacy to receive a treatment with certain attributes: no increased risk of secondary malignancy in the future (−16.3% efficacy; 95% CI 16.1% to 16.5%), a treatment with a fixed duration with IV/SC administration at the clinic (−5.2%; 95% CI 3.9% to 6.1%), and a treatment containing targeted therapy (−5.8%; 95% CI 5% to 6.4%). Conversely, patients would only accept a treatment with the side effect of neuropathy or atrial fibrillation if they would receive 7.2% (95% CI −11.3% to −4.3%) and 1.4% (95% CI −4.3% to 0.7%) additional treatment efficacy in return respectively (Figure 4).

### 3.4 Additional analyses

The preferences observed in the total group of patients were not statistically different across subgroups according to the covariates' gender, age, and previously treated status. Due to technical issues in the online questionnaire, the question on gender did not show up in a majority of the online surveys resulting in a large number of missing data for this variable. In a sub‐analysis in patients where the variable gender was present (*n* = 65), gender did not have a significant effect on treatment preference. Educational level, however, did influence patient preferences (*p* = 0.014), with regards to slight differences in the preference for adverse events **(**Table S1 **)**.

### 3.5 Patients' caregivers' preferences

For caregivers, only 3 out of 5 attributes had significant influence on their treatment preferences. These attributes were 5‐year PFS, risk of secondary malignancy, and type of agent. Their preferences within these attributes did not differ significantly from the patients (*p* = 0.22). The caregivers preferred a treatment with higher 5‐year PFS, containing targeted therapy with no increased risk of secondary malignancy in the future. Type of adverse events and duration and setting of treatment had no significant influence on the preferences of the caregivers of WM patients (Figure S1).

## 4 DISCUSSION

Shared decision‐making, which is a key component of patient‐centered health care, necessitates a good understanding of WM patients' priorities regarding their treatment. We, therefore, developed a Dutch language DCE to investigate this further. All attributes included in the DCE significantly influenced the patient preferences for WM treatment.

Specifically, WM patients prioritized a long PFS and a low risk of secondary malignancies. Indeed, these are both relevant issues in a disease that is incurable, yet comes with a long overall survival. With regard to side effects, patients disliked being at risk of neuropathy the most, even more than nausea, vomiting, and extreme fatigue, and were willing to trade 6.5% efficacy to avoid neuropathy. This underscores the importance of timely dose reduction in patients experiencing neuropathy following treatment with proteasome inhibitors and/or the choice for less neurotoxic alternatives.^25^ Also, it illustrates the need to search for active regimens that are not neurotoxic. Patients are 8% more likely to choose a treatment with IV/SC agents in the hospital with a fixed duration over a long‐term treatment with an oral agent at home. This is also consistent with their willingness to accept lower efficacy to receive a fixed‐duration treatment. It may seem surprising that patients would prefer IV/SC administration at hospital over oral intake at home, but this could be explained by the fixed duration of the IV/SC treatment, which patients seem to value the most. We did not test the attribute “duration of treatment” separately since the oral treatments currently available for WM do not have a fixed duration. Interestingly, fixed‐duration treatments for WM usually contain cytotoxic drugs that predispose for secondary malignancies, a risk that respondents also wanted to avoid. Modern targeted drugs such as BTK inhibitors are not associated with secondary malignancies but need to be used continuously until progression. Thus, our results demonstrate the need to develop effective, non‐neurotoxic WM treatment regimens without cytotoxic agents but with a fixed duration. Such regimens are currently not available for WM. However, targeted therapies with fixed duration have been successfully explored in other hematological malignancies such as chronic lymphocytic leukemia and chronic myeloid leukemia.^26^, ^27^, ^28^

Patient preference data can provide valuable information for directing future clinical trials, the development of novel drugs, and clinical guidelines and can also support health‐care decision‐making including approval of novel drugs. Insight into which characteristics and outcomes of treatment are most important to patients can be helpful to clinicians in supporting shared decision‐making with their patients. Fortunately, research on how to incorporate patient preferences into health‐care decision‐making is expanding.^29^

Compared to patients, caregivers included fewer attributes into their decisions and side effects did not significantly affect their preferences. Thus, caregivers seemed to give more weight to the efficacy of the treatment compared to the adverse effects. Awareness of this discrepancy might be helpful in discussions on treatment options with patients and their families.

This is, to the best of our knowledge, the first study evaluating patient preferences in WM. Patient preferences in other hematological malignancies such as multiple myeloma (MM) and chronic lymphocytic leukemia (CLL) have been studied.^30^, ^31^, ^32^, ^33^ Both MM and WM patients value PFS as the most important attribute but differ on their appraisal of mode of administration. Among relapsed/refractory MM patients, an all‐oral regimen was preferred, as opposed to the preference of WM patients for an IV/SC treatment in our study.^30^ However, in the study with MM patients, the duration of the treatment (fixed vs ongoing) was not included in the attribute levels while in the current study IV/SC was merged with fixed duration. In patients with CLL, a disease with great resemblance to WM with regards to various biologic and clinical characteristics, the same combination of mode and duration of treatment was assessed. Interestingly, patients with CLL seemed to prefer an oral agent taken indefinitely over an IV treatment for the duration of 6 months. However, it was the attribute with the least influence on treatment preference and when out‐of‐pocket costs were added to the DCE, the IV treatment (which had lower out‐of‐pocket costs compared to oral treatment) was preferred.

Although treatment‐naïve versus previously treated status did not influence patients' treatment preferences in our study, it could be that the number of therapy lines would have demonstrated potential differences, however we did not collect those data. Sociodemographic characteristics did not influence patient preferences with the exception of educational level. Educational level only influenced preference for type of adverse event. However, this subanalysis had insufficient power to determine the exact differences between high and low educational level as more respondents are needed to answer this question. A possible explanation is that educational level influences the medical knowledge of patients, thereby allowing patients to consider the long‐term consequences of certain side effects. This might suggest that extra care has to be taken to counseling of patients with a lower education regarding adverse events.

Strengths of our study include the careful selection of attributes and levels by involving WM experts as well as patients in developing the protocol. Also, we reached a relatively large sample size for this rare disease since the annual incidence and prevalence of WM in the Netherlands are approximately 350 and 1200 patients, respectively, and 214 patients completed the survey.^34^

Limitations of the current study include the possibility of selection bias, as the online survey was published on the website of the patient organization and thus could have reached a certain subset of patients only. Also, we were concerned that older patients could be less active online and would therefore be less likely to participate. Still, the median age of the study participants was 66 years, which approaches the median age of WM patients at diagnosis based on population‐based cancer registry data in the Netherlands (70 years).^12^ The educational level of the respondents corresponded to that of the general population.^35^ Patients rated the difficulty of the survey with a median of 5.4 out of a 10‐point scale. However, based on the patient interviews in the pilot study, this was mostly because they found the questions emotionally challenging rather than finding the questions difficult to comprehend or complex. We, therefore, do not feel that the DCE task itself was too difficult. Finally, we are not certain these results are generalizable to WM patients worldwide because of differences in culture, infrastructure, and health‐care systems. For example, in the current study, we did not include treatment costs as an attribute since cancer care in the Netherlands falls under the basic health‐care insurance which is fully covered by all insurance providers precluding co‐pay options. Also, the Netherlands is densely populated and patients rarely have to travel great distances for a treatment at the hospital.

In conclusion, we present the first systematic data on WM patient preferences based on treatment characteristics. We found that WM patients find efficacy (high 5‐year PFS rate) the most important attribute, followed by a low risk of future secondary malignancies. Neuropathy was the adverse event they most wanted to avoid. Patients preferred a fixed‐duration IV/SC treatment with targeted therapy as opposed to chemotherapy over an ongoing oral regimen. These data can be supportive in discussions with individual patients about their treatment preference. Also, the results suggest that future clinical trials in WM should focus on highly effective, non‐neurotoxic regimens without cytotoxic drugs and with a fixed duration.

## AUTHOR CONTRIBUTIONS

KA, JV, and PN designed the study; MJK, MM, and JV provided assistance with patient recruitment; KA and PN analyzed the data; PN provided statistical support; KA wrote the manuscript with contributions from all authors, who also interpreted the data, and read, commented on, and approved the final version of the manuscript.

## CONFLICT OF INTEREST

The authors declare no significant competing financial, professional, or personal interests that might have influenced the performance or presentation of the work described in this manuscript.

## ETHICS APPROVAL

The study protocol was submitted to the Ethical Committee at Amsterdam University Medical Centers, but since the survey was anonymous and did not contain personal data, the need for formal ethical approval and informed consent was waived in accordance with Dutch legislation.

## Supporting information


Figure S1

Table S1

## Data Availability

The datasets used during the current study are available from the corresponding authors on reasonable request.
