# Supplementary figures and images for: Patient preferences regarding treatment options for Waldenström's macroglobulinemia: A discrete choice experiment
Source: Cancer Med. 2022 Jul 26;12(3):3376–86. doi: 10.1002/cam4.5080 (PMC9939214; doi:10.1002/cam4.5080)

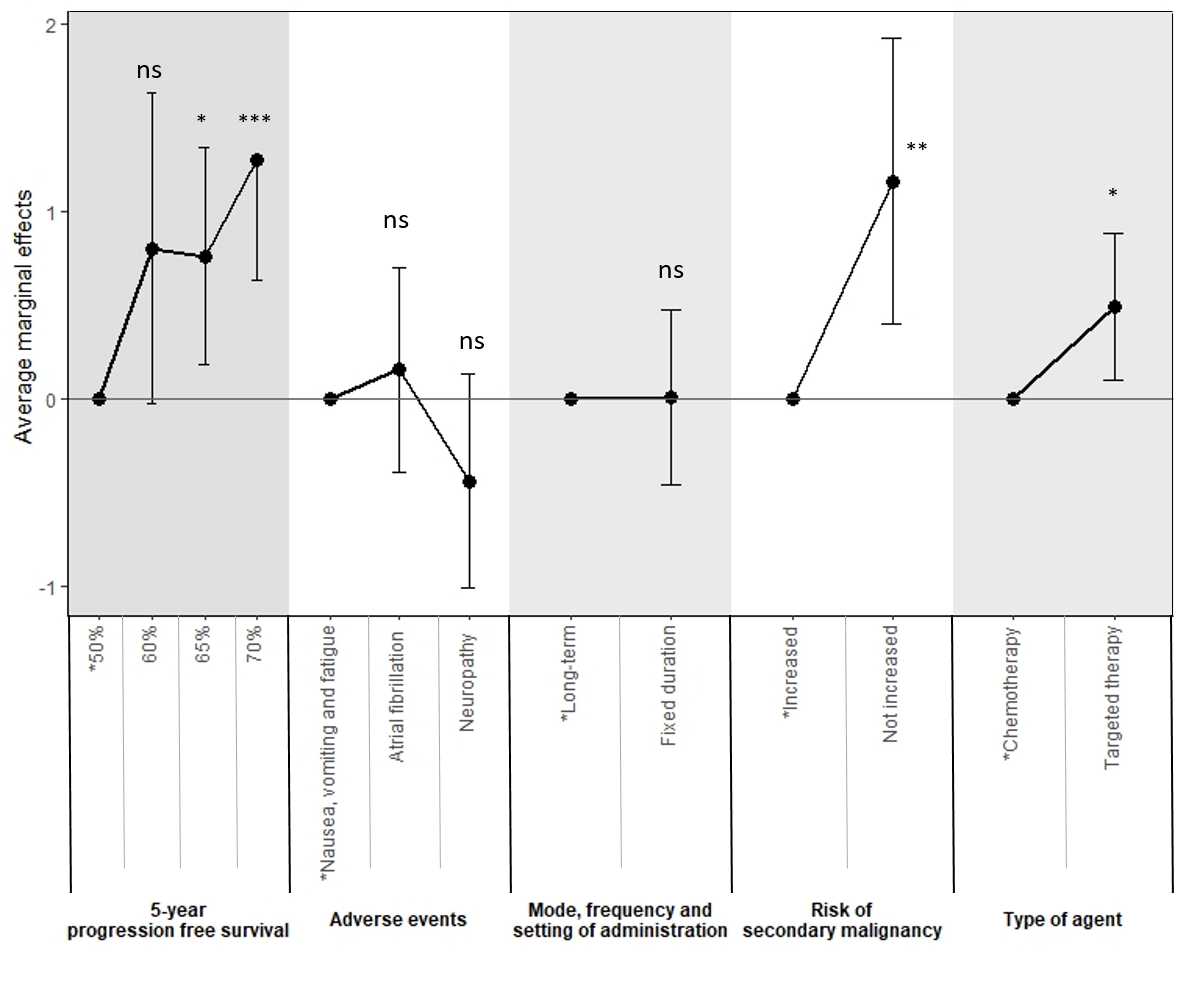

Supplement: Supplementary file 1 — Figure S1 [file CAM4-12-3376-s001.tif]
