# Supplementary material for: Patient preferences regarding treatment options for Waldenström's macroglobulinemia: A discrete choice experiment
Source: Cancer Med. 2022 Jul 26;12(3):3376–86. doi: 10.1002/cam4.5080 (PMC9939214; doi:10.1002/cam4.5080)
Supplement: Supplementary file 2 — Table S1 [file CAM4-12-3376-s002.docx]

**Supplemental Table S1.** Marginal effects of high education level and lower education level patient preferences for WM treatment

| **Attributes and levels** | **Higher education level** | **Lower education level** | **P value** |
| --- | --- | --- | --- |
| Number of patients | 115 | 94 | 0.014 |
| 5-year progression free survival: | 0.29 (0.25 to 0.34) | 0.24 (0.18 to 0.29) | - |
| Mode, frequency and setting of administration: |  |  |  |
| Long-term (reference)  Fixed duration | 0.08 (0.04 to 0.11) | 0.07 (0.03 to 0.12) | - |
| Adverse events: |  |  |  |
| Nausea, vomiting and fatigue (reference)  Neuropathy  Atrial fibrillation | -0.1 (-0.14 to -0.05)  0.05 (0.001 to 0.09) | -0.10 (-0.15 to -0.05)  -0.10 (-0.15 to -0.05) | -  - |
| Risk of secondary malignancy: |  |  |  |
| Increased (reference)  Not increased | 0.22 (0.16 to 0.27) | 0.22 (0.16 to 0.29) | - |
| Type of agent: |  |  |  |
| Chemotherapy (reference)  Targeted therapy | 0.08 (0.05 to 0.11) | 0.08 (0.05 to 0.11) | - |
